# Rapid Evaporation of Water on Graphene/Graphene-Oxide: A Molecular Dynamics Study

**DOI:** 10.3390/nano7090265

**Published:** 2017-09-07

**Authors:** Qibin Li, Yitian Xiao, Xiaoyang Shi, Shufeng Song

**Affiliations:** 1 Chongqing Key Laboratory of Heterogeneous Material Mechanics, College of Aerospace Engineering, Chongqing University, Chongqing 400044, China; sfsong@cqu.edu.cn; 2 Key Laboratory of Low-Grade Energy Utilization Technology & System, Ministry of Education, College of Power Engineering, Chongqing University, Chongqing 400044, China; 20123656@cqu.edu.cn; 3 Department of Earth and Environmental Engineering, Columbia University, New York, NY 10027, USA; xs2144@columbia.edu

**Keywords:** graphene, graphene-oxide, water, evaporation, molecular dynamics simulation

## Abstract

To reveal the mechanism of energy storage in the water/graphene system and water/grapheme-oxide system, the processes of rapid evaporation of water molecules on the sheets of graphene and graphene-oxide are investigated by molecular dynamics simulations. The results show that both the water/graphene and water/grapheme-oxide systems can store more energy than the pure water system during evaporation. The hydroxyl groups on the surface of graphene-oxide are able to reduce the attractive interactions between water molecules and the sheet of graphene-oxide. Also, the radial distribution function of the oxygen atom indicates that the hydroxyl groups affect the arrangement of water molecules at the water/graphene-oxide interface. Therefore, the capacity of thermal energy storage of the water/graphene-oxide system is lower than that of the water/graphene system, because of less desorption energy at the water/graphene-oxide interface. Also, the evaporation rate of water molecules on the graphene-oxide sheet is slower than that on the graphene sheet. The Leidenfrost phenomenon can be observed during the evaporation process in the water/grapheme-oxide system.

## 1. Introduction

The development of modern society is accompanied by severe problems such as energy crises, environmental pollution and greenhouse effects [1], etc. Enhancing the efficiency of energy utilization is one of the effective ways to ease these problems. Using nanofluids to improve the thermophysical properties of working fluid is an ideal approach to optimize energy utilization [2,3]. Studies have reported [4,5,6,7] that the thermal conductivity of working fluid can be enhanced by adding nanoparticles. As a novel working medium, nanofluids have great applications in the fields of energy, biochemistry, automotive, construction, aerospace and so on [8,9,10,11].

Also, the unique mechanism of energy storage of nanofluids has been proposed. A large amount of fluid molecules is able to adhere to the surface of nanoporous materials due to the attractive fluid–solid interaction. More energy is needed to desorb the fluid molecules from the surface of nanoporous materials. Therefore, this kind of nanofluid will store more energy than pure working fluid via the adsorption/desorption processes. Chen et al. [12,13] performed extensive research on the energy absorption of nanofluids in carbon nanotube under the actions of heat, force, and electric coupling fields. McGrail et al. [14] proposed using the metal-organic heat carrier nanofluids to improve the efficiency of the organic Rankine cycle. The novel working nanofluids have the potential to increase the power output by up to 15%.

One method to enhance the energy storage in nanofluids is to increase the surface area of nanomaterials. Graphene and graphene-oxide are the novel carbon-based nanomaterials which can be considered as the ultimate case of the family of flat polycyclic aromatic hydrocarbons. The specific surface area of graphene and graphene-oxide is ultra large, and the thermal conductivity of graphene is up to 5200 W/(m∙K) [15]. Therefore, graphene based materials have great prospects in the field of energy storage based on the absorption/desorption process at the fluid–solid interface.

So far, the reported experiments about phase transition of fluid on graphene based materials have focused on nucleation or condensation. Kimmel et al. [16] observed a metastable ice on graphene by experiments and ab initio molecular dynamics simulations. Severin et al. [17,18] investigated the properties of condensed water and water/ethanol in the grapheme–mica slit by scanning force microscopy. Algara-Siller et al. [19] found a novel structure of ice (square ice) in graphene nanosheets based on experiments and molecular dynamics (MD) simulations. Zheng et al. [20] observed the ice nucleation on graphite. Bampoulis et al. [21,22,23,24] studied the ice and water/alcohol in graphene/mica/MoS_2_ slits by atomic force microscopy.

Here, the processes of rapid evaporation of water molecules on the sheets of graphene and graphene-oxide are investigated by MD simulations [25]. The work is also expected to provide useful insights into the microstructures of water molecules [26].

## 2. Model and Computational Method

MD is a method which can describe the movement, structure and geometry of molecules by classical mechanics. MD has been proven to be an efficient tool to reveal the micro/nano-mechanisms of novel materials.

### 2.1. Simulation Model

**Figure 1:**
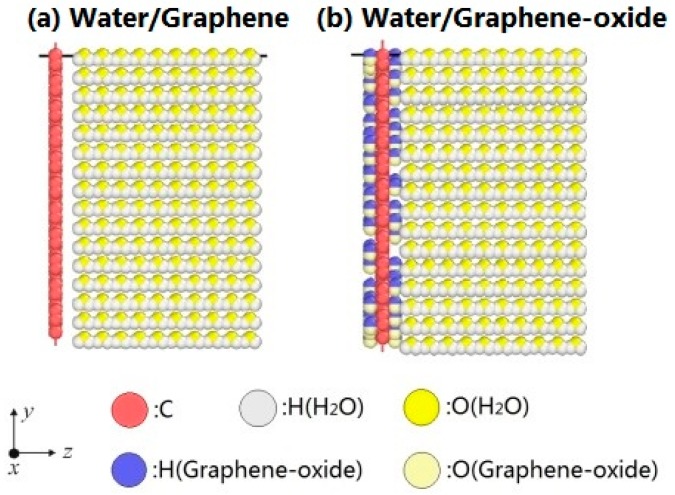
Evaporation models. (**a**) Water/Graphene system; (**b**) Water/Graphene oxide system.

The configurations of water molecules on graphene and graphene-oxide are shown in Figure 1. The configurations of atoms are visualized by the open software OVITO [27]. The water/graphene system is composed of a single graphene layer (880 carbon atoms) and 2560 water molecules. The size of the simulation box is *X*: 0–50 Å, *Y*: 0–50 Å, *Z*: 0–115 Å. The water molecules form a thin film on the *XOY* plane. The system expands in the direction of the *Z* axis during evaporation. Similarly, the water/grapheme-oxide system is composed of a single graphene-oxide layer (880 carbon atoms and 81 hydroxyl, –OH) and 2560 water molecules, which are in a simulation box of the same size. As noted, experiments have shown that the hydroxyl groups and epoxy groups are randomly distributed on a monolayer of graphene-oxide [20]. Here, the monolayer of graphene-oxide is only constructed by carbon atoms and hydroxyl groups for simplicity [28].

### 2.2. Modeling Parameters

MD simulations are performed by using LAMMPS (large-scale atomic molecular massively parallel simulator) [29]. The OPLS [30] (Optimized Potentials for Liquid Simulations) potential is employed to describe the interactions between particles,

(1) $$E(\phi )=E_{nb}(\phi )+E_{bond}(\phi )+E_{angle}(\phi )+E_{torsion}(\phi )$$

where *E_nb_*(*φ*) is the non-bonding energy, *E_bond_*(*φ*) is the stretching energy, *E_angle_*(*φ*) is the bending energy, *E_torsion_*(*φ*) is the dihedral energy. The details of these energies are shown as follows,

(2) $$E_{bond}=\sum_{bonds}k_{r}{\left(r-r_{eq}\right)}^{2}$$

(3) $$E_{angle}=\sum_{angles}k_{\theta }{\left(\theta -\theta_{eq}\right)}^{2}$$

where *k_r_* is the elastic constant of the bond, *r_eq_* is the equilibrium bond length, *k_θ_* is the bond angle bending elastic constant, *θ_eq_* is the equilibrium angle.

(4) $$E_{nb}=\sum_{i}^{on\;a}\sum_{j}^{on\;b}\left\{q_{i}q_{j}e^{2}/r_{ij}+4\varepsilon_{ij}\left[{\left(\frac{\sigma_{ij}}{r_{ij}}\right)}^{12}-{\left(\frac{\sigma_{ij}}{r_{ij}}\right)}^{6}\right]\right\}$$

where $q_{i}$,$q_{j}$ are the charges of two particles, *r_ij_* is the distance between two atoms, *ε_ij_* is the potential energy, *σ_ij_* is the length parameters.

(5) $$\begin{array}{ll} E_{torsion} & =\sum_{i}\frac{V_{1}^{i}}{2}\left[1+\cos(\phi_{i}+f_{i}1)\right]+\frac{V_{2}^{i}}{2}\left[1-\cos(2\phi_{i}+f_{i}2)\right]\\ & +\frac{V_{3}^{i}}{2}\left[1+\cos(3\phi_{i}+f_{i}3)\right]+\frac{V_{4}^{i}}{2}\left[1-\cos(4\phi_{i}+f_{i}4)\right] \end{array}$$

$V_{1-4}^{i}$ is the distortion constant of the dihedral angle, *Φ_i_* is the dihedral angle, *f_i_* is the phase angle. Note the phase angle is 0 in the model. The potential parameters of the studied particles in the manuscript are shown in Table 1.

Periodic boundary conditions are applied to the *X*, *Y* and *Z* directions. The Nose–Hoover algorithm [31] controls the temperature of the system. The cutoff radius is 12 Å and the timestep is 0.1 fs in the simulations. Initially, the systems in Figure 1 are equilibrated at 300 K for 1,000,000 steps in NVT ensemble. Then, the systems are heated to 1000 K in 1,000,000 steps. Next, the systems are maintained at 1000 K for 1,000,000 steps. The three simulation periods are denoted as 300 K, 300–1000 K, and 1000 K in the next section. Also, the water/graphene system and water/graphene-oxide system are denoted as “G system” and “GO system”, respectively.

In addition, a pure water system (i.e., the G system in Figure 1a without the layer of graphene) is also simulated, which experiences the same simulation periods (300 K, 300–1000 K, and 1000 K). The energy changes during the process of evaporation in the pure water system are compared with the changes in the G system and the GO system.

## 3. Results and Discussion

### 3.1. Distribution of Water Molecules

**Figure 2:**
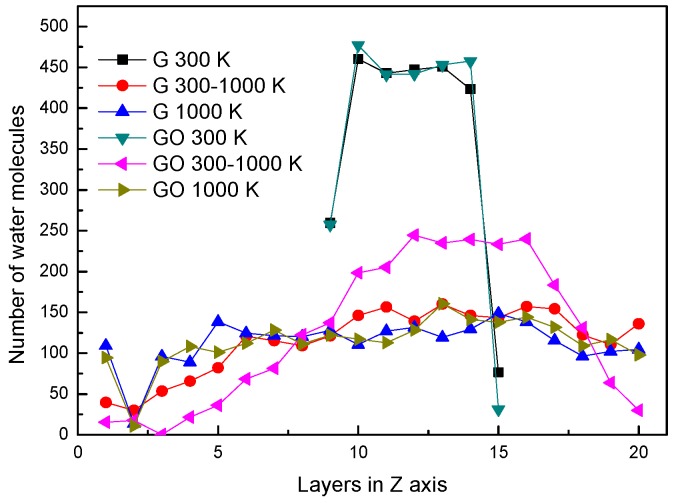
Distribution of water molecules in the *Z*-axis.

In the two systems, the distributions of water molecules during the end of each simulation period in the *Z* direction are shown in Figure 2. The simulation box is divided into 20 layers along the *Z* direction, in order to analyze the properties of water molecules. At the conditions of 300 K and 1000 K, the distributions of water molecules along the *Z* direction are similar in the G system and GO system. At the condition of 300 K, the water molecules clustered as a liquid state in several layers at the end of the simulation. At the condition of 1000 K, the water molecules distribute uniformly in the simulation box at the end of the simulation. This means that the water molecules in both systems have already evaporated. Note that at 300–1000 K, the number of water molecules in the layers of 10–14 in the GO system is larger than that in the G system. This indicates that a large amount of water molecules are still agglomerated in the GO system. In other words, the amount of evaporated water molecules in the GO system is less than that in the G system during the end of the heating process at 300–1000 K.

**Figure 3:**
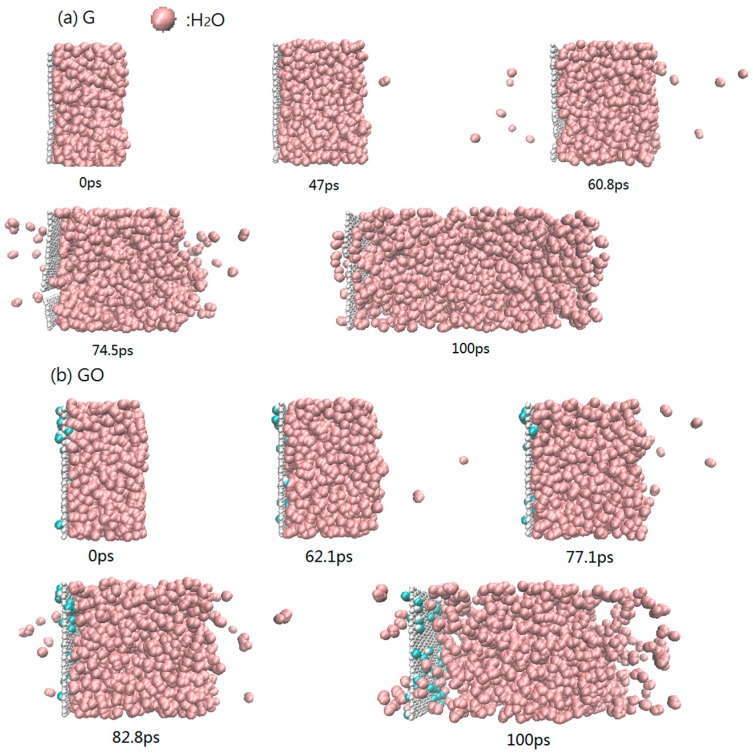
Configuration of atoms during the heating process at 300–1000 K (**a**) G system; (**b**) GO system.

The snapshots of the heating process during 300–1000 K in the G system and GO system are shown in Figure 3. During the heating process, the first evaporated water molecule is observed at 47.0 ps in the G system, while the other is observed at 62.1 ps in the GO system. As the heating continues, more water molecules evaporate from both systems. At the end of the simulation, the Leidenfrost phenomenon [32] is obviously observed in the GO system: the water clusters are separated from the surface of grapheme-oxide sheet. The phenomenon is due to the interaction force between the fluid and solid. Because the interaction force between water molecules and the sheet of graphene is larger than that between water molecules and the sheet of grapheme-oxide, the Leidenfrost phenomenon is only observed in the GO system. The existence of hydroxyl groups in the GO system could increase the distance between water molecules and the carbon atoms on the surface of grapheme-oxide. The enlarged distance will reduce the attractive force between the carbon atoms of the grapheme-oxide sheet and the water molecules. In other words, the existence of hydroxyl groups reduces the adsorption capacity of the grapheme-oxide sheet. Therefore, in the evaporation process, the water molecules on the surface of grapheme-oxide will be separated from the surface of the grapheme-oxide sheet, resulting in the Leidenfrost phenomenon which could slow the evaporation rate of the water molecules.

### 3.2. Radial Distribution Function

**Figure 4:**
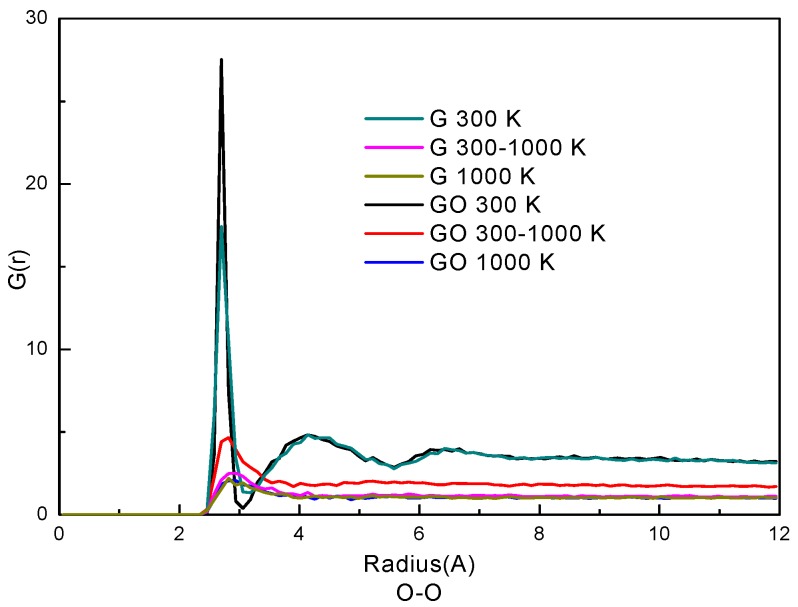
Radial distribution function (RDF) of O–O atoms.

The radial distribution function (RDF) represents how the density of a particle varies as a function of distance from a reference particle. Usually, RDF is used to analyze the phase state of particles in a system. Here, the RDFs of oxygen atoms of water molecules in the two systems are plotted in Figure 4. The similarity of the curves in the G system and the GO system indicates that the trends of phase transition of the water molecules are similar in the two systems. At the condition of 300 K, the RDFs have two peaks in both systems. The first peak of the RDF is at 2.82 Å and the second peak is at 4.41 Å in both systems. The positions of the two peaks are in accordance with our previous study of liquid water [26,33]. Also, the RDFs indicate that water molecules at 300 K are in a denser state than those at 300–1000 K and 1000 K; the RDFs of 300–1000 K and 1000 K only have one peak which indicates that the water molecules are in gaseous form. By comparing the RDFs in the two systems, the G(r) in the GO system is larger than that in the G system at the corresponding simulation period, especially at the condition of 300–1000 K. This indicates that the water molecules are denser and more agglomerative in the GO system at 300–1000 K. Therefore, the phase transition in the GO system needs more time to break the network of hydrogen bonds among water molecules, thereby reducing the evaporation rate.

### 3.3. F3 Order Parameter of Water Molecules

It has been reported [34] that the water molecules are orderly arranged in the microscopic form which can be expressed by the order parameter. Although the order parameter of water molecules is usually applied to analyze condensed water molecules, the *F*_3_ order parameter is employed to investigate the difference of microstructures of water molecules in the G system and the GO system during the evaporation process. The *F*_3_ consists of a three-body configuration, which indicates that the degree of tetrahedral arrangement is formed by any one oxygen atom and other oxygen atoms around it, defined as,

(6) $$F_{3}=\langle {\left(\cos\varphi_{jik}\left|\cos\varphi_{jik}\right|+\cos^{2}\left(109.47^{\circ }\right)\right)}^{2}\rangle$$

where *φ_jik_* is the angle between the specified oxygen atom *i* and the other two oxygen atoms around it; 109.47° is the angle of H–O–H. For the crystal structure of water (hydrate and ice), the oxygen atom and the surrounding four oxygen atoms in water molecules form a saturated hydrogen bond. The *F*_3_ is zero in the whole system, while *F*_3_ is 0.1 in the case of liquid water.

**Figure 5:**
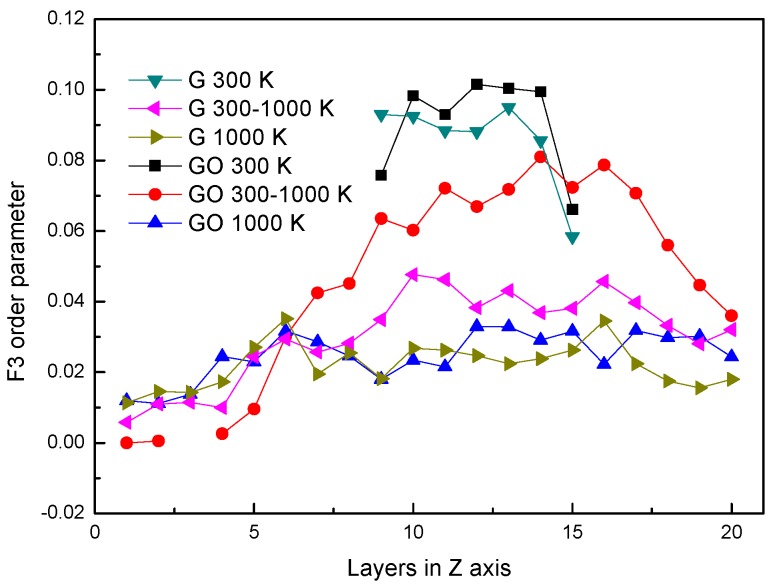
*F*_3_ order parameter.

In Figure 5, the distributions of *F*_3_ in different systems are similar to Figure 2. The distributions of *F*_3_s are almost the same at 300 K in both systems. However, at the same period, the *F*_3_ in the GO system is smaller than that in the leftmost part of the G system. It shows that the existence of hydroxyl groups could influence the arrangement of oxygen atoms in water molecules at the fluid–solid interface. The *F*_3_s have little difference in both systems after evaporation.

### 3.4. Energy Storage in the Systems

Theoretically, the thermal energy storage in the novel nanofluids consists of three parts: (1) the phase transition energy of the working fluid, (2) the energy change of the solid nanoparticles during heating, and (3) the desorption energy at the fluid–solid interface. The total energy change of the system during evaporation is regarded as the sum of the three energies. The total energy change in the G system (*E*_G_) and the GO system (*E*_GO_) are compared in Table 2. Note that the energy change in the pure water system is designated as *E*_Water_. The results show that both the G system and GO system can store more energy than the pure water system, and the G system can store more energy than the GO system. This is because hydroxyl groups in grapheme-oxide can reduce the adsorption capacity between the sheet of grapheme-oxide and water molecules, which results in lower desorption energy at the fluid–solid interface.

## 4. Conclusions

The rapid evaporation processes of water molecules on the graphene and graphene-oxide sheets were studied by molecular dynamics in this paper. The results draw the following conclusions:

The water molecules in the water/graphene system evaporate faster than that in the water/graphene-oxide system. The water molecules are denser in the water/graphene-oxide system. The Leidenfrost phenomenon can be observed in the water/graphene-oxide system, which is caused by hydroxyl groups on the surface of graphene-oxide. The hydroxyl groups also result in the disorder arrangement of the oxygen atoms in water molecules in the water/graphene-oxide system. Both the water/graphene system and the water/graphene-oxide system can store more energy than the pure water system. However, the existence of hydroxyl groups will reduce the desorption energy at the fluid–solid interface and result in less energy storage in the water/graphene-oxide system than that in the water/graphene system.

## Figures and Tables

**Table 1:** Optimized Potentials for Liquid Simulations (OPLS) potential parameters of particles [28].

Graphene	Graphene Oxide	H_2_O
$\varepsilon_{C=C}=285.1$	$\varepsilon_{C=C}=285.1$	$\varepsilon_{O=O}=650.3$
$\sigma_{C=C}=3.3997$	$\sigma_{C=C}=3.3997$	$\sigma_{O=O}=3.166$
$r_{C=C}=1.4000$	$\varepsilon_{O=O}=650.3$	$\varepsilon_{H-O}=0$
$k_{r,C=C}=1.963\times 10^{6}$	$\sigma_{O=O}=3.166$	$\sigma_{H-O}=0$
$\theta_{C=C=C}=120$	$\varepsilon_{C=O}=392.0$	$r_{O-H}=1$
$k_{\theta }=2.69\times 10^{5}$	$\sigma_{C=O}=3.190$	$k_{r,O-H}=1.884\times 10^{6}$
	$r_{C=O}=1.4100$	$q_{H}=0.424$
	$k_{r,C=O}=1.340\times 10^{6}$	$q_{O}=-0.848$
	$r_{O-H}=0.9450$	$\theta_{H-O-H}=109.47$
	$k_{r,O-H}=2.315\times 10^{6}$	$k_{\theta ,H-O-H}=2.302\times 10^{5}$
	$\theta_{C=C=C}=120$	
	$k_{\theta ,C=C=C}=2.69\times 10^{5}$	
	$\theta_{C=O-H}=108.5$	
	$k_{\theta ,C=O-H}=2.30\times 10^{5}$	
	$\theta_{C=C=O}=109.5$	
	$k_{\theta ,C=C=O}=2.093\times 10^{5}$	

Note: Units of the parameters: *ε* (J/mol); *σ* (Å); *r* (Å); *k_r_* (J/(mol·Å^2^)); *k_θ_* (J/(mol·rad^2^)); *θ* (°); *q* (e).

**Table 2:** The comparison of energy change in different systems.

Ratio	Value
*E*_G_/*E*_Water_	1.18
*E*_GO_/*E*_Water_	1.06
